# The use of 3D-printed models in patient communication: a scoping review

**DOI:** 10.2217/3dp-2021-0021

**Published:** 2022-01-19

**Authors:** Gemma Traynor, Andrew IU Shearn, Elena G Milano, Maria Victoria Ordonez, Mari Nieves Velasco Forte, Massimo Caputo, Silvia Schievano, Hannah Mustard, Jo Wray, Giovanni Biglino

**Affiliations:** 1 Bristol Medical School, University of Bristol, Bristol, BS8 1UD, UK; 2 Great Ormond Street Hospital for Children, NHS Foundation Trust, London, WC1N 3JH, UK; 3 University Hospitals Bristol & Weston, NHS Foundation Trust, Bristol, BS1 3NU, UK; 4 Institute of Cardiovascular Science, University College London, London, WC1E 6DD, UK; 5 National Heart & Lung Institute, Imperial College London, London, SW3 6LY, UK

**Keywords:** 3D models, 3D printing, communication, rapid prototyping

## Abstract

3D models have been used as an asset in many clinical applications and a variety of disciplines, and yet the available literature studying the use of 3D models in communication is limited. This scoping review has been conducted to draw conclusions on the current evidence and learn from previous studies, using this knowledge to inform future work. Our search strategy revealed 269 papers, 19 of which were selected for final inclusion and analysis. When assessing the use of 3D models in doctor–patient communication, there is a need for larger studies and studies including a long-term follow up. Furthermore, there are forms of communication that are yet to be researched and provide a niche that may be beneficial to explore.

## Plain language summary

3D models can be made out of different materials and can copy a patient’s own body, for example, a patient’s heart. Various studies have been conducted which have explored how these unique 3D models can be used to aid communication between doctors and patients. This paper aims to identify the available studies on this topic published in the medical literature and summarize their findings in order to guide future research. A total of 19 papers covering different specialties were included and summarized in this review. Upon summarizing these papers, it was noticed that more studies are needed with a large number of participants, and that the researchers need to follow up these participants over time to see if their opinion on using 3D models for communication changes. Studying communication between patients and their family and friends may also be useful to fully appreciate the usefulness of 3D models in this context.

## Graphical abstract

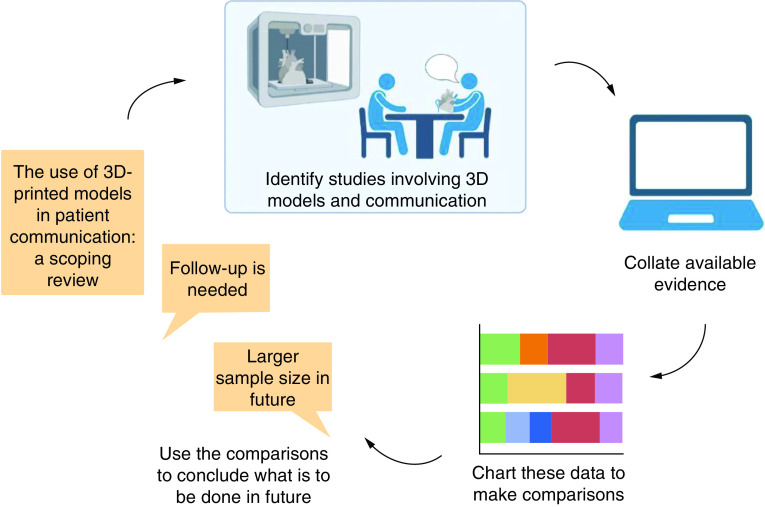

## 3D modeling

In the last decade, 3D models have been employed in a variety of applications such as decision-making, surgical planning, trainee education and more recently, communication. 3D printing can be used to construct individual models that are unique to a patient’s anatomy. The literature on the use of 3D models in the medical field is now extensive; however, there are very few papers on the use of 3D models in communication.

## Communication

It is generally well accepted that effective doctor–patient communication leads to overall better health outcomes. A plethora of studies have found that improving doctor–patient communication leads to increased adherence to treatment, a more effective recovery and better emotional health following discharge [1–3]. Additionally, the relationship between a doctor and a patient can have an impact on the length of hospital stay and the number of complications, in turn, affecting the cost analysis associated with each patient [4,5]. There may be a variety of reasons for these observations. Having effective communication between doctors and their patients means that the patients are more likely to understand their illness and comprehend the potential consequences of not following their treatment plan [6]. Furthermore, a relationship of trust is often built in order to have effective communication and this leads to patients feeling more comfortable to ask questions and engage in the consultation. Patient engagement within a consultation has been shown to influence the style in which a doctor conducts a consultation [7]. It was shown that patients that are most engaged and responsive during their consultation receive more patient-centered care from physicians [8]. Patient-centered care has been the focus of clinical practice since medicine has evolved to be less focused on a biomedical model and shifted more to a psycho-biomedical model. This shift in clinical practice, and thus in doctor–patient relationships, was also associated with increased patient autonomy, with patients no longer being passively treated and, instead, being involved with decision-making and being empowered to take control of their health.

## Aim of scoping review

Given the abundance of evidence of effective communication increasing patient satisfaction, some research has focused on how to improve communication during clinical practice [9–12]. Therefore, this review aims to analyze the available literature within all medical disciplines and use the current evidence to map this niche and inform future work. This review aims to answer the following question: what is the role of 3D models in doctor–patient communication? No other reviews have been conducted to analyze this aspect. A scoping review has been deemed the most appropriate methodology to follow due to limited literature available and in light of the aims that have been set out, following considerations by Armstrong *et al.* [13]. A scoping review can be seen as complimentary to other types of reviews, and although conducting this scoping review could act as a precursor to a systematic review, this is not its purpose. There is variability in the conduct of scoping reviews, however, the decision to conduct this review for the sole aim of mapping this broad topic is in line with guidance from a number of authors [14,15].

## Methods

This scoping review was conducted following the Joanna Briggs Institute (JBI) methodology without the optional sixth stage [16]. The key steps were identification of the research question, identification of relevant studies, study selection, charting the data and finally, collating, summarizing and reporting the results.

### Search strategy

A comprehensive review of the available literature was conducted with no limitation on the date the literature was published. This was done in order to gather detail on all of the available literature as of April 2021. The search strategy used included: (((((3D model[Title/Abstract]) OR (3D modeling[Title/Abstract])) OR (3D modeling[Title/Abstract])) OR (3D printing[Title/Abstract])) OR (3D models[Title/Abstract])) AND (Communication[Title/Abstract]). The results were reviewed for the inclusion criteria reported below.

### Databases

PubMed and Embase were used to identify any relevant papers. Due to the nature of the research behind 3D modeling and communication potentially having a psychological aspect, Embase was used to broaden the search for relevant papers. These results were then reviewed individually. Any relevant papers were charted onto a table and cross-referenced with the PubMed results.

### Inclusion criteria

Inclusion criteria were defined before conducting the review and were detailed as follows:

- Studied communication (i.e., direct assessment of interaction and communicative dynamics among specialists or between specialists and nonspecialists, including patient understanding)
- Original research
- Full studies (no conference abstracts)
- Written in English

### Exclusion criteria

Exclusion criteria were based on the inclusion criteria and were refined during the process of data collection. This was in line with guidance from Armstrong *et al.* on how to conduct a scoping review, stating that inclusion and exclusion criteria may be adapted as data are collected [13]. This differs from a systematic review in which inclusion and exclusion criteria must be defined from the onset of the process. Here, exclusion criteria included:

- Mentioned the word communication but in a different context (e.g., ‘communication between cells’)
- Review, editorial or conference abstract
- Nonhuman subjects
- Paper was about 3D bioprinting
- Communication was not an objective

### Data charting

Two researchers independently assessed the abstract of each unique paper identified by the searches to determine whether they met criteria for inclusion. If it was ambiguous whether a paper should be included, the full article was read and discussed by the two researchers in order to decide whether to include or exclude it. This process was documented using an Excel spreadsheet. This included results of the search strategy and whether each paper was included or excluded along with an exclusion category code.

A data extraction tool was then constructed including: PMID, first author, discipline, type of study, themes, aim, general results, results specific to communication, type of condition, how data were collected, whether the data were qualitative or quantitative, the type of participant(s), type of communication, whether study participants were randomized, the sample size and finally, whether there was a follow up for studying communication. Results were color coded according to medical disciplines (e.g., cardiovascular, orthopedic etc.) to facilitate comparisons.

### Logic model

Common themes identified across the papers were analyzed and mapped in a visual representation, creating a logic model to graphically encapsulate the theory of how the intervention (i.e., 3D models) produces its outcomes (Figure 3). First, components that were consistently occurring with the use of the models were identified. Subsequently, causal mechanisms were identified from these components, in other words, those observations that were noted as a result of the components associated with the model. Lastly, the end results were established from the literature; these outcomes included enhanced communication, building a rapport and benefiting patient education. Mapping the stages that led to the outcomes that were observed allows us to learn from the available literature how and why these models can be an asset to enhancing communication.

## Results

### Specialties

**Figure 1.**
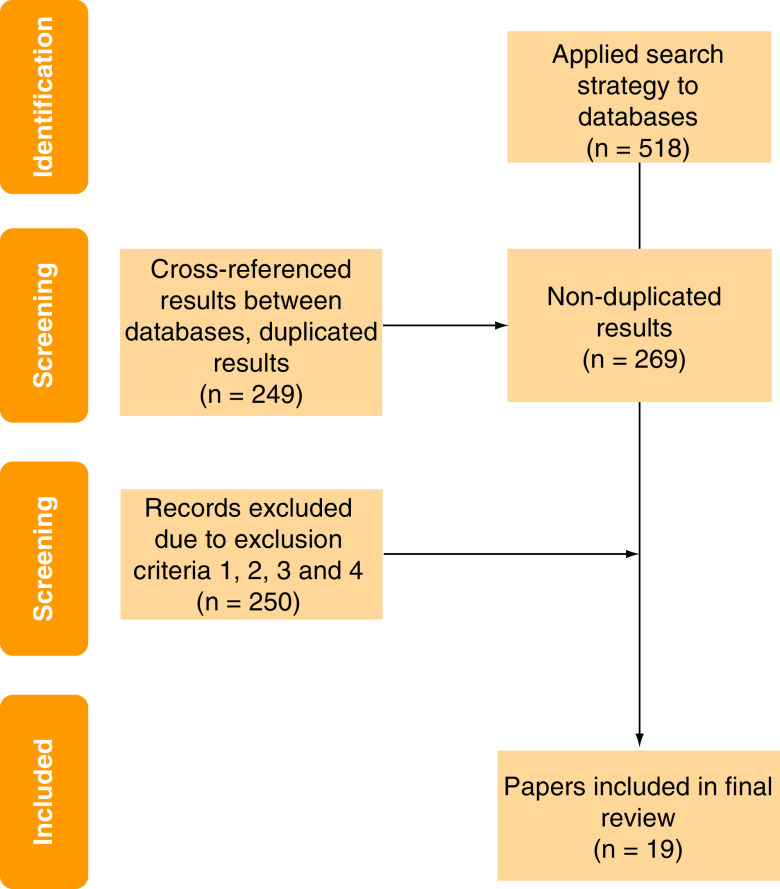
CONSORT diagram of the identification of papers from the search strategy to the final inclusion of results.

**Figure 2.**
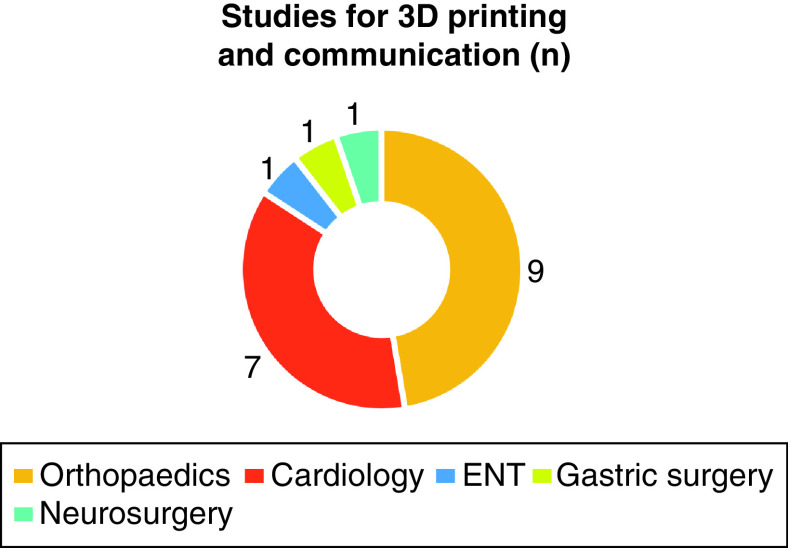
Specialties of the final included results.

The overall search strategy revealed 518 papers. After cross-referencing for duplicates, 269 papers were analyzed. Following the application of the exclusion criteria, 19 papers ultimately qualified for inclusion (Figure 1). The earliest paper was from 1991, however, 91% of the literature dated from 2013 onward. These included papers from within the field of orthopedics (n = 9), cardiology and cardiovascular surgery (n = 7), ear nose and throat (n = 1), gastric surgery (n = 1) and neurosurgery (n = 1) (Figure 2 & Table 1).

**Table 1.** Details of which diseases have been studied by the included papers.

Orthopedics	Cardiology	ENT	Gastric surgery	Neurosurgery
Complex fractures	Hypertrophic obstructive cardiomyopathy	Thyroid cancer	Colon cancer	Glioma
Trimalleolar fracture	Congenital heart defects			
Pilon fractures				
Articular fracture				
Humeral intercondylar fractures				
Complex elbow fractures				
Calcaeal fractures				
Tibial plateau fractures				
Bone tumors				

ENT: Ear, nose and throat.

### Study design

There was heterogeneity in the study design. For the orthopedic studies, seven out of nine were randomized (i.e., model vs nonmodel), one was a cross-sectional study and another was a case series. Only one cardiac paper was a randomized study, four were cross-sectional studies, one was a case report and another was a cross-over trial in which the patients acted as their own controls. The one ear nose and throat paper included was a cross-sectional study, the one neurosurgery study included was also a cross-sectional design and lastly, the gastric surgery study that was included followed a randomized design procedure.

### Sample size

The sample size of the 19 studies ranged from n = 1 participant (case report) to n = 103 participants in one randomized cardiac study. The median sample size across all of the disciplines was n = 50 (interquartile range [IQR]: 55). The median sample size for the cardiac studies was n = 34 (IQR: 49), while for the orthopedic papers it was n = 74 (IQR: 50).

### Data collection

Seventeen studies used questionnaires to gather feedback about the models and two of these also included interviews. Of the two studies that did not use questionnaires, one used semistructured interviews and the other collected verbal feedback via telephone or email.

None of the studies included a follow up on the use of the 3D models in communication. Some followed-up patients for health outcomes and only one mentioned that a follow up could be useful to assess the longer term outcomes in communication, but it was not included in the published study. The questionnaire-based studies used rating scales to evaluate the models’ features and, in some cases, complimented these observations by collecting free text comments which were qualitatively analyzed (n = 9).

### Communication

The main type of communication studied was patient–doctor communication, with studies in the pediatric setting also considering communication between clinicians and families. Three cardiac studies evaluated communication between colleagues, while other disciplines looked solely at doctor–patient communication. There was variation in the type of participant included with a mix of studies evaluating patients, parents, clinicians and trainees and a combination of these. The majority of the noncardiac studies solely analyzed patients for assessing 3D models in communication and did not record a clinician’s perspective.

### Feedback on models

In all of the 19 studies feedback and results on the use of 3D models for communication were positive, irrespective of the type of respondent or type of communication. Illmann *et al.* found that of the 85% of clinicians who found benefit from the models, 80% of them believed they would facilitate communication with colleagues and 72% believed they would be useful in communication with parents or families [17]. In an orthopedics study, patients were asked ‘How much does the CT or 3D-printing model help you to gain a better communication with doctors?’ [18] and patients in the 3D-printing group rated the 3D-printed model an average of 8.5 compared with 6.5 for those rating the CT scans. Some patients found the 3D models to be particularly useful when used in conjunction with other modalities, for example, when viewed alongside radiological images [19]. A minority of studies produced findings on communication that were not exclusively positive. One study asked clinicians to rank which applications they felt the models would work best in, the clinicians ranked teaching as the most relevant and communication as the least relevant [20]. Another study reported patients having to emotionally confront the model as a barrier to its utility when faced with their brain tumors [19]. Further detail on the results of all 19 studies is shown in Supplementary Table 1.

**Figure 3.**
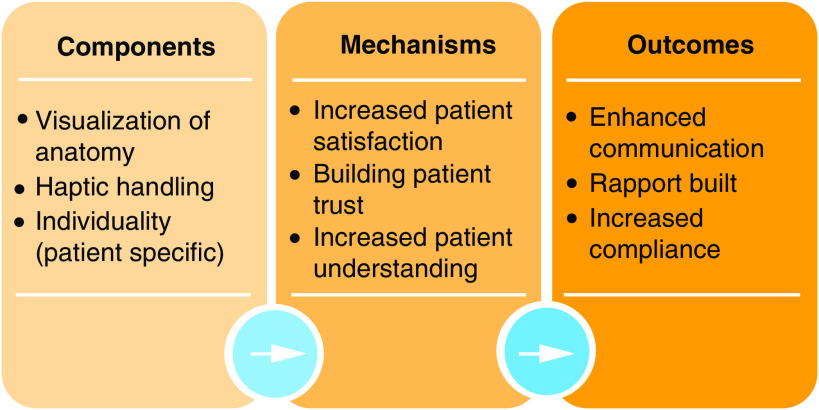
Logic model for the use of 3D models for communication in medicine.

A logic model was created and is shown in Figure 3. Key components for the use of 3D models for communication purposes include the ability of visualizing the anatomy, the patient-specific nature of the model and the opportunity for haptic handling.

## Discussion

3D printing holds promise to assist and facilitate communication in clinical practice. Considering the broad search strategy without limitation on the date of publication and the broad literature on 3D printing in medicine, results of this search revealed a very modest number of studies focused on communication, highlighting that this is an underexplored topic which is still in its infancy. Most of the analyzed literature dated from 2013 onward, which demonstrates the considerable expansion of the world of 3D printing in the last decade. The reasoning behind the lack of emphasis on the communication aspect that 3D printing offers is not entirely clear. Possible reasons include lack of guidance on how to analyze this application of 3D printing, as well as the time pressure which is typical of clinical commitments. This review may help to identify successful approaches and to recognize that this is an area with many opportunities for novel and exciting research.

### Study characteristics

The sample size of the studies varied both between and within the disciplines, likely due to the differences in recruitment, participant type and experimental procedure. There were some challenges in analyzing the sample sizes of the studies due to the different recruiting strategies. For example, some of the studies did not specify how many clinicians were surveyed for analysis of communication, and so there was some disparity in whether clinicians were included within the sample size, or their observations were anecdotally reported.

Many of the studies did not use a comparator to be able to effectively draw conclusions as to whether 3D models are more or less effective in communication. Instead, these studies chose to gauge opinions on 3D models using, for example, a cross-sectional study design. This research could act as building blocks for further research to be conducted with a more pragmatic methodology, including a randomization procedure. Having these studies as a base for future work is useful in that the positive feedback gained on 3D models may encourage others to experiment with the 3D-printing technology and explore its versatilities in different types of communication. Although all of the studies received positive feedback for the 3D models, none of the studies included a follow up. Many of the studies followed-up patients for other objectives, such as comparing surgical approaches and therefore, measuring health outcomes after a sufficient time period has passed. There was only one paper [19] that mentioned the possibility of following up the patients’ thoughts on communication months after the initial consultation. This could be seen as a potential weakness in the design of many studies as the interplay between a patient’s emotional well-being and physical health is a well-established phenomenon that can have powerful implications [21]. Patients may have had positive feelings toward the 3D models initially when first introduced to the concept but in the succeeding months, these feelings may shift. This could be, for example, due to growing feelings of anxiety when visualizing their own anatomy. This was recognized by Biglino *et al.*, who found that 30% of their sample patients reported feeling more anxious when confronted with the model [22]. Including a follow up in future studies is therefore important to monitor the evolution of these dynamics, ideally engaging patients in the process.

Reviewing the literature from a variety of disciplines has meant that comparisons can be made between them, and lessons can be extracted from one field of medicine and utilized in another. This was the case with one paper by van de Belt *et al.*, which studied patient education and communication with 3D models for glioma treatment [19]. This was a unique paper in that it focused on potential psychological effects that may come with the first experience of a patient-specific 3D model. This paper revealed both positive and negative psychological effects in this experience, many other papers failed to acknowledge due to reducing communication to patient understanding. Instead of examining the effects of the model on the patient holistically, many papers chose to focus on how much the patients can learn about their anatomy and condition and neglected to perceive that this experience may be daunting for many patients and that many patients may not want to know as much detail as a 3D model can provide.

### Quantitative & qualitative assessment

The majority of the studies collected quantitative data; this was done most commonly via a Likert scale on a questionnaire to be filled out by the participant. There are advantages and disadvantages to both quantitative and qualitative data, however, what is imperative to remember is the complexity of communication. Communication represents a key part of human behavior and assessing and conveying beliefs on a 1–10 scale is a reductionist approach that negates acknowledgment of the dynamic nature of communication. Furthermore, the empirical nature of scales may cause them to be labeled as objective; however, these scales are inevitably subjective. Despite the issues associated with the gathering of communication data via Likert scales, they hold their place in some contexts. For example, future work could collect quantitative data alongside qualitative data (e.g., via interview or free-text comments), an approach adopted in three of the 19 studies.

### Communication as the primary focus & different facets of communication

Although all of the included studies studied communication, assessing communication was not a primary objective for the majority. Most chose to assess different uses of 3D printing, mainly surgical planning, and measured objective health outcomes. Often communication was only analyzed using a questionnaire that included one or two questions about communication with the 3D models among other questions (not communication based). Therefore, the actual number of studies that focused on communication was actually less than 19. There is a need for future studies to evaluate the use of models in communication as a sole or primary objective. There should be a research effort placed on focusing on communication as highlighted by the almost universally positive results observed by the 19 studies.

Most of the analyzed literature stated in their objectives that communication will be the focus of the study; however, the observations they recorded were often focused on patient education. An example of this would be when researchers asked patients ‘How much do you understand about your condition?’ and ‘How much do you know about the surgical plan?’ as in [23]. Extrapolating improved communication dynamics from increased patient understanding may be misleading as although these may intertwine, they are different entities. While increased patient understanding is desirable and may suggest effective communication, communication should not be assessed exclusively on patient knowledge and understanding. Furthermore, many of the papers assessed communication by simply asking ‘Do you *feel* the models facilitated communication?’, and while direct assessment is useful, it may be more valuable alongside assessing communication indirectly. An example of a more meaningful assessment of communication could be to present more open questions allowing participants to share their experiences more freely, how they use the model in the own time if they are given one to take home, or how the haptic handling of the model guided the consultation and helped them to engage more in the discussion.

### Limitations

This scoping review was limited to PubMed and Embase and did not consult other databases, or gray literature. Also, studies not published in English were excluded. Due to the limited literature that has been published on 3D models and communication, a systematic review with a meta-analysis could not be conducted. It was therefore decided that a scoping review was more appropriate, recognizing the limitations of this but also the value of mapping the available literature to inform suggestions for future work.

## Conclusion

This scoping review was undertaken because of the lack of any assessment of published studies on the use of 3D models for communication and that future work would benefit from guidance. Recommendations stemming from this review can inform the design of future studies exploring the use of 3D patient-specific models in facilitating communication. We argue that this is a clinically relevant area of research, with potentially important implications for patient empowerment and psychological adjustment.

## Future perspective

Doctor–patient communication was the most frequently studied form of communication, with all of the noncardiac papers studying this type of communication. Although this review included studies which only included clinicians, these studies assessed clinicians’ opinions on how 3D models affect communication with patients. This review did not include any studies which solely assessed communication between specialists. Some of the cardiac papers studied communication between parents/families and physicians; however, overall, the range of communication that has been studied is limited. Specifically, no studies were identified where the use of 3D models in communication between patients and their family and friends was the focus. This could be an interesting area to explore in future work as participants in some of the included studies mentioned utilizing the models to better explain their condition (or their child’s condition) to relatives and friends. As a patient’s diagnosis, especially a life-long diagnosis, is often a large part of a patient’s life, the ability to effectively communicate this with those whom they are closest to could be invaluable. Compared with patient–doctor communication (i.e., between a specialist and a nonspecialist), this is a case of communication between nonspecialists, as relatives and friends may have very little knowledge of normal anatomy. The 3D models could add value to patients’ lives further than just in the consultation room.

One area that has not been investigated is whether the use of models is more beneficial in communication with individuals with certain diagnoses. For example, within the cardiac context, six out of the seven studies looked at models of congenital heart disease. The other study looked at hypertrophic obstructive cardiomyopathy. Despite congenital heart disease encompassing a range of diagnoses, there was no direct comparison of whether any of the single diagnoses received greater benefit from the models. This may have been due to the limited sample size not allowing for reliable subgroup analyses and the uniqueness of each patient’s heart when it comes to complex anatomical defects. Additionally, congenital heart disease could be compared with other types of structural pathologies such as cardiomyopathies or valve disease. These comparisons between diseases could guide where 3D printing is best suited to be implemented, whether with patients with congenital heart disease who tend to be younger, or patients with valvular disease who tend to be older. If positive feedback is received from a range of patients with varying diagnoses this would only strengthen the case for implementing 3D models into clinical practice to ultimately improve patient care.

Much of the research that is currently available is of limited sample size and often not randomized. In the future, this area of research can progress further by conducting larger-scale randomized studies, comparing the use of 3D models to an effective comparator such as conventional procedures. Furthermore, this research should include both qualitative and quantitative data collection in which a broader sense of patient perspective about the models can be ascertained. In doing this, richer data can be gathered including any emotional and psychological effects the participants may feel toward the models, which will, in turn, affect communication. Additionally, research into how models can be used by patients to communicate with friends and family about their condition would be a novel approach and would provide an insight into how 3D models can be employed in patients’ lives.

It will be interesting to evaluate the role of the models in aiding communication not only across disciplines but also during specific milestones in the patient’s journey, such as transition into adulthood, pregnancy and end of life.

**Figure 4.**
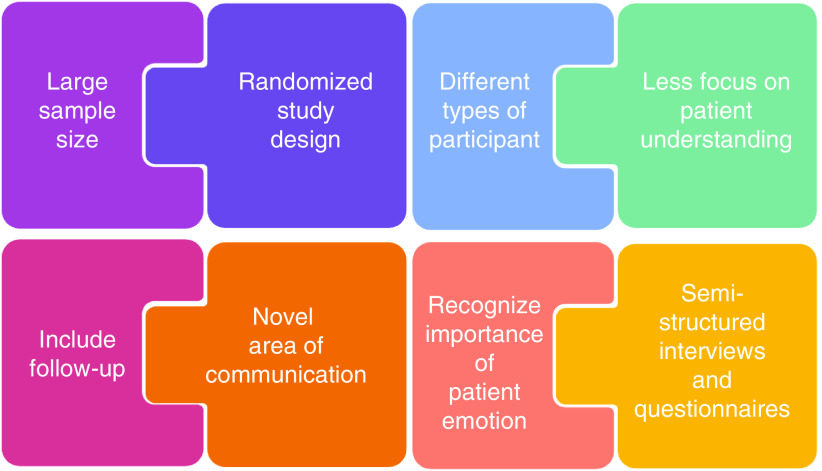
Summary of suggestions for study design of future research.

Based on the evaluation of these studies, future studies could benefit from incorporating these suggestions in their study design. These suggestions have been summarized in Figure 4. Studies should include a large sample size which will be dependent on the pathology which is being studied, as well as utilizing a randomized study design including different types of participants and collecting opinions from specialists and nonspecialists. There should be a shift in focus away from solely evaluating patient understanding, and more toward the assessment of patient emotion and its effect on communication. A follow up should be included. Communication between nonspecialists is a new area to explore. Semistructured interviews with a smaller population could be used to gather more detailed and in-depth perceptions alongside questionnaires with the larger population.

**Executive summary**

**Objective**

- To chart the available data from studies on the use of 3D models in communication and analyze these data to draw conclusions and inform future work.

**Methods**

- A PubMed and Embase search was conducted with a comprehensive search strategy from which papers were identified and analyzed in detail.
- Comparisons were made between studies to identify strengths and weaknesses in both protocol and results.

**Results**

- A total of 269 papers were identified from which 19 papers were deemed relevant and included in the final analysis.
- The majority of papers had positive outcomes for communication.

**Discussion**

- Larger randomized studies are needed with communication as a primary objective.
- These studies should include a follow up to observe results over time.
- Communication between nonspecialists is an interesting concept that is yet to be explored.
