# Supplementary material for: The use of 3D-printed models in patient communication: a scoping review
Source: J 3D Print Med. 2022 Jan 19;6(1):13–23. doi: 10.2217/3dp-2021-0021 (PMC8852361; doi:10.2217/3dp-2021-0021)
Supplement: Supplementary file 1 [file 3dp-06-13-s1.docx]

| **Study Title** | **Study Design** | **Sample Size** | **Communication Outcomes** | **Other Outcomes** |
| --- | --- | --- | --- | --- |
| The efficacy of using 3D printing models in the treatment of fractures: a randomised clinical trial [23] | Randomised control trial | 48 patients  30 surgeons | Mean score for ‘how much would you like the doctor to use a 3D prototype to communicate with you about your condition?’ = 9.3 ± 0.5  Mean score for ‘usefulness of the 3D prototype for communication with patients’ = 9.1 ± 0.8. | Treatment of complex fractures using 3D printing reduced the frequency of intraoperative fluoroscopy, blood loss volume and operative time, but did not improve postoperative function compared with routine treatment. |
| Application of 3D Printing in the Surgical Planning of Trimalleolar Fracture and Doctor-Patient Communication [24] | Randomised control trial | 30 patients | Patient satisfaction = 9.3 ± 0.6 points.  3D-printing can faithfully reflect the anatomy of the fracture site, effectively help doctors plan the operation and physician-patient communication. | Fracture prototypes were accurate, with an relatively high score of verisimilitude and effectiveness. Operation time and intraoperative blood loss in 3D printing group were less than those in non-3D printing group (P < 0.05). |
| The Feasibility of 3D Printing Technology on the Treatment of Pilon Fracture and Its Effect on Doctor-Patient Communication [18] | Randomised control trial | 100 patients | ‘How much does the CT or 3D printing model help you to gain a better communication with doctors?’ = 8.5 for 3D printed model vs. 6.5 for CT scan (mean score).  ‘Overall assessment of the conversation with CT or 3D printing model’ = 9 for 3D printed model vs. 7.5 for CT scan (mean score).  ‘How much does the 3D printing model help you to communicate with patients?’ = 8.8 ± 1.0 (mean score). | 3D printing group showed significantly shorter operation time, less blood loss volume and fluoroscopy times (all *P* < 0.001), higher rate of anatomic reduction (*P* = 0.040) and rate of excellent and good outcome (*P* = 0.029) than CT group |
| Value of three-dimensional printing of fractures in orthopaedic trauma surgery [25] | Cross-sectional Study | 52 patients | Mean patient comprehension score increased from 6.26 ± 1.26 to 8.21 ± 0.84 after introduction of the 3D models, with average increase of 1.95 (+31.15%, P<0.05). Use of the models improved patient trust in the proposed treatments and most patients (88%) favoured the routine use of 3D-printing for explaining interventions (Yes 46, No 6). | 3D-printed replicas of articular fractures facilitated surgical planning and preoperative simulations, as well as training and teaching activities. They also strengthened the informed consent process and reduced surgical times and costs by about 15%. |
| Application of 3D-printing technology in the treatment of humeral intercondylar fractures [26] | Randomised Control Trial | 91 patients | Average score from patients was 8.8 ± 1.0 when asked ‘How much does the 3D-printing model help you to gain a better communication with doctors?’  Average score from doctors was 8.7 ± 0.9 when asked ‘How much does the 3D-printing model help you to communicate with patients?’ | Operation duration, blood loss volume and fluoroscopy times for 3D-printing group was 76.6 ± 7.9 minutes, 231.1 ± 18.1 mL and 5.3 ± 1.9 times, and for conventional group was 92.0 ± 10.5 minutes, 278.6 ± 23.0 mL and 8.7 ± 2.7 times, respectively. |
| Three-dimensional printing technology and materials for treatment of elbow fractures [27] | Randomised Control Trial | 40 patients | The average mean scores for patients who were asked to rate ‘(1) the overall assessment of the conversation with CT or 3D model’ and to rate (2) the usefulness of the CT or 3D model for understanding the medical condition were 9 (3D printing group) compared to 6.7 (conventional control group) and 8.7 (3D printing group) compared to 6.2 (conventional control group) respectively. | The 3D group showed shorter surgical duration, lower blood loss and higher elbow function score, compared with the conventional group |
| Comparison of the Conventional Surgery and the Surgery Assisted by 3d Printing Technology in the Treatment of Calcaneal Fractures [28] | Randomised Control Trial | 75 patients | The questionnaire from doctors showed that the overall satisfaction and usefulness of the 3D printing models were high.  The questionnaire from patients also exhibited high scores of overall satisfaction with the use of a 3D printing model.  For patients, 3D printed models are able to help them better understand their conditions and preoperative plan and facilitate the communication with doctors. | The operation duration, blood loss volume and number of intraoperative fluoroscopy for 3D printing group was 71.4 ± 6.8 minutes, 226.1 ± 22.6 ml and 5.6 ± 1.9 times, and for conventional group was 91.3 ± 11.2 minutes, 288.7 ± 34.8 ml and 8.6 ± 2.7 times respectively. There was statistically significant difference between the conventional group and 3D printing group (*p* < 0.05). |
| Comparison of traditional surgery and surgery assisted by three dimensional printing technology in the treatment of tibial plateau fractures [29] | Randomised Control Trial | 72 patients | Mean score for patients asked on ‘The effect of using 3D models to communicate with your doctors’ was 8.2 ± 0.2.  Mean score for doctors asked ‘How critical is the 3D printing model in helping your communication with patients?’ was 8.6 ± 0.1. | Average operation time, average amount of blood loss, and number of intra-operative fluoroscopy for 3D model group was 85.2±0.9 minutes, 186.3± 5.5ml, 5.3± 0.2 times, and for traditional surgery group was 99.2±1.0 minutes, 216.2 ±6.9 ml,7.1 ± 0.2 times respectively. There was statistically significant difference between the traditional surgery group and 3D model group (P < 0.05) |
| Combining Augmented Reality and 3D Printing to Improve Surgical Workflows in Orthopedic Oncology: Smartphone Application and Clinical Evaluation [30] | Case-series | 6 patients  9 surgeons | The mean average score for clinicians when asked to rate 3D printed models in combination with augmented reality for the use in patient combination was 4.76 out of 5. | The results and the positive feedback obtained from surgeons and patients suggest that the combination of AR and 3D printing can improve efficacy, accuracy, and patients’ experience. |
| Application of 3D printing in the surgical planning of hypertrophic obstructive cardiomyopathy and physician-patient communication: a preliminary study [31] | Cross-sectional Study |  | Patients highly appreciated the role of 3D model in preoperative conversations and the overall satisfaction score was 9.11±0.38 points.  Mean patient score were asked ‘Would you like the doctor to use a 3D-printed prototype to communicate with you about your condition?’ was 9.40±0.49 | The 3D-printed prototypes were accurate and useful for preoperative evaluation, surgical planning, and practice. |
| 3D-manufactured patient-specific models of congenital heart defects for communication in clinical practice: feasibility and acceptability [32] | Randomised Control Trial | 103 parents | Parents and cardiologists both found models to be very useful in discussing congenital heart defects. Parental knowledge was not associated with their level of education (p=0.2) and did not improve following their visit. Consultations involving 3D models lasted on average 5 min longer (p=0.02).  Feedback included:   - The model can stimulate curiosity (ie, better engagement than other media), especially in youngsters; and - 3D models might be especially helpful at the time of the initial diagnosis, combined with a reference model (ie, normal cardiac anatomy). |  |
| Involving patients, families and medical staff in the evaluation of 3D printing models of congenital heart disease [20] | Cross-sectional Study | 13 patients  15 parents  14 clinicians  11 nurses | Stakeholders’ responses suggested that models can have a role in communicative processes. Their patient-specific quality is important and patients indicated that 3D models can help them visualise ‘what’s going on inside’. Parents agreed that models can spark curiosity in young people.  Patients specifically spoke about the value of the models in enhancing their understanding of heart conditions. Reportedly models were ‘clearer than CMR scans’. | Clinicians ranked teaching as the most relevant potential application of 3D models, while communication was ranked as least relevant.  Nurses agreed that 3D models improved their learning experience during a CHD course. |
| Utility and Access to 3-Dimensional Printing in the Context of Congenital Heart Disease: An International Physician Survey Study [17] | Cross-sectional Study | 47 Clinicians | Some 85% (60/71) agreed or strongly agreed that patient-specific 3D cardiac models are or can be a beneficial tool in treating patients with CHD. The leading perceived benefits of the 3D models were that they facilitated communication with colleagues (80.0%, 48/60) or with patients and their families (72%, 43/60). Only 3% of respondents disagreed or strongly disagreed that 3D models were beneficial. | The primary reason for lack of access was financial barriers (50%, 18/36). In clinical practice, surgical planning is the primary use of models (96%, 26/27), followed by interventional catheterization planning (52%, 14/27). Double outlet right ventricle was the most commonly modelled lesion (70%, 19/27). |
| Clinical value of patient-specific three-dimensional printing of congenital heart disease: Quantitative and qualitative assessments [33] | Cross-sectional Study | 2 cardiac surgeons  2 cardiologists | All reported that models would be invaluable in improving the consultation experience as they would be able to explain the CHD to the patients more efficiently. Patients could understand the condition of their heart better with the use of 3D printed model. However, when being asked whether the consultation time could be reduced, only two agreed. | Models were also deemed helpful for planning procedures and for teaching applications |
| Piloting the Use of Patient-Specific Cardiac Models as a Novel Tool to Facilitate Communication During Clinical Consultations [22] | Cross-over trial | 20 patients | The majority of participants reported that 3D models were fun and useful for their understanding, improving their visit, yet 30% participants indicated that the model made them feel anxious about their condition. Nevertheless, participants reported that they would want to have a 3D model for future visits, and they would recommend it to a peer. Improvements in confidence, knowledge, narrative and patient experience suggest value of 3D models in communication. |  |
| The Perception of a Three-Dimensional-Printed Heart Model from the Perspective of Different Stakeholders: A Complex Case of Truncus Arteriosus [34] | Case report | 1 patient | The father of the patient commented very positively on the 3D model. “[The surgeon] present[ed] us with the 3D model […] and used that to help and explain exactly what he needed to do and why he needed to do it, so it [was] really fascinating to see it, really helpful. The first thing I noticed was how big it was, and I hadn’t comprehended how big the heart was, […] how it actually fitted in [my son]. To be able to turn it round and notice how the pulmonary arteries were narrowed, […] to actually see it in front of you it was fantastic.” | Positive remarks from the parents, patients, surgeons, cardiologist, imager and trainee. |
| Patient-Specific Actual-Size Three-Dimensional Printed Models for Patient Education in Glioma Treatment: First Experiences [19] | Cross-sectional Study | 11 patients | Model improved patients' understanding about their situation; patients reported that it was easier to ask questions using a model and that it supported their decision about preferred treatment. A perceived barrier for using the 3D model was that it could be emotionally confronting, particularly in an early phase of the disease. Positive effects were related to psychological domains, including coping, learning effects, and communication. | 3D models helped patients to understand the location and size of the tumour, and provided a clearer image than 2D images seen on the neurosurgeon's screen. Other patients stated that the situation became even clearer when the 3D model was used in combination with radiologic images on the screen. |
| Usefulness of three-dimensional printing of superior mesenteric vessels in right hemicolon cancer surgery [35] | Randomised Control Trial | 61 patients | 95% patients in the 3D printed model group rated communication as ‘effective’ compared to 68.4% in the 3D image group and 63.6% in the control group.  General satisfaction was higher in the 3D printed group compared to the 3D image group, and control group with scores of 85%, 73.7% and 72.7% respectively. | The duration of surgery for the 3D printing and 3D image groups was significantly reduced (138.4 ± 19.5 and 154.7 ± 25.9 min vs. 177.6 ± 24.4 min, P = 0.000 and P = 0.006), while the number of lymph node dissections for these 2 groups was significantly increased (19.1 ± 3.8 and 17.6 ± 3.9 vs. 15.8 ± 3.0, P = 0.001 and P = 0.024) compared to the control group. |
| Usefulness of a 3D-Printed Thyroid Cancer Phantom for Clinician to Patient Communication [36] | Cross-sectional Study | 33 patients  10 clinicians | 90% of clinicians rated the 3D model as ‘very effective’ for clinician-explained procedures for thyroid surgery, with the other 10% still rating the model as ‘effective’.  Patient understanding increased with the use of 3D model, without the use of the 3D models 24% of patients rated the effectiveness of understanding the relationship of structures around the thyroid gland as ‘not clear’, 35% said it was effective and 41% said it was ‘very effective’.  Once they were shown the 3D model 18% stated it was effective for understanding the relationship of structures around the thyroid gland, with the other 82% saying it was ‘very effective’. |  |

**Supplementary Table 1.** Summary of studies included in the final analysis, including communication outcomes and other outcomes not related to communication. None of studies reported follow-up data. Colour legend (disciplines): yellow = orthopaedics; red = cardiovascular; blue = ENT; green = gastric surgery; aqua = neurosurgery.
